# Assessment of nursing care using indicators generated by software^1^

**DOI:** 10.1590/0104-1169.0177.2547

**Published:** 2015

**Authors:** Ana Paula Souza Lima, Tânia Couto Machado Chianca, Meire Chucre Tannure

**Affiliations:** 2 MSc, RN, Hospital da Polícia Militar, Belo Horizonte, MG, Brazil; 3 PhD, Full Professor, Escola de Enfermagem, Universidade Federal de Minas Gerais, Belo Horizonte, MG, Brazil; 4 PhD, Adjunct Professor, Escola de Enfermagem, Pontifícia Universidade Católica de Minas Gerais, Belo Horizonte, MG, Brazil

**Keywords:** Nursing Process, Intensive Care Units, Quality Indicators, Health Care, Software Validation

## Abstract

**OBJECTIVE:**

to analyze the efficacy of the Nursing Process in an Intensive Care Unit using indicators generated by software.

**METHOD:**

cross-sectional study using data collected for four months. RNs and students daily registered patients, took history (at admission), performed physical assessments, and established nursing diagnoses, nursing plans/prescriptions, and assessed care delivered to 17 patients using software. Indicators concerning the incidence and prevalence of nursing diagnoses, rate of effectiveness, risk diagnoses, and rate of effective prevention of complications were computed.

**RESULTS:**

the Risk for imbalanced body temperature was the most frequent diagnosis (23.53%), while the least frequent was Risk for constipation (0%). The Risk for Impaired skin integrity was prevalent in 100% of the patients, while Risk for acute confusion was the least prevalent (11.76%). Risk for constipation and Risk for impaired skin integrity obtained a rate of risk diagnostic effectiveness of 100%. The rate of effective prevention of acute confusion and falls was 100%.

**CONCLUSION:**

the efficacy of the Nursing Process using indicators was analyzed because these indicators reveal how nurses have identified patients' risks and conditions, and planned care in a systematized manner.

## Introduction

A search for improved quality of care provided to patients has gained prominence in the world context in recent decades and nursing, as well as in other professions, has faced the need to improve work processes^(^ 1 ^)^.

The Systematization of Nursing Care (SNC) is indicated as a method capable of improving the quality of nursing care because it provides scientific support, security and direction to the performance of the nursing staff's activities^(^ 2 ^-^ 3 ^)^. One of the tools that should be used in the implementation of SNC is the Nursing Process (NP), a scientific method nurses can use to apply technical-scientific and practical knowledge into clinical practice^(^ 4 ^)^. Its effective application leads to improved healthcare quality, encourages the construction of evidence-based theoretical and scientific knowledge, helps in the development of protocols, grounds teaching and clinical rationales, and the management of costs and planning of resource allocation to quality nursing services^(^ 5 ^-^ 6 ^)^.

The increased volume of data accruing from recording NP stages favors the development and broader use of computer systems, which enables the pursuit of actions to be directed based on organized data that becomes available to professionals through nursing information systems^(^ 7 ^)^.

The computerized system with Nursing Process in Intensive Therapy (SIPETi) consists of software based on the Theory of Basic Human Needs, especially designed by Wanda de Aguiar Horta for adult Intensive Care Units (ICUs). It contains essential data for the recording of investigations, diagnoses, planning, implementation and assessment of the delivery of nursing care, in addition to the results and indicators of such care. SIPETi was experimentally applied in an ICU to establish its applicability. Data, however, were not sufficient to generate all indicators concerning nursing care delivery proposed in the system's assessment module.

Given the need to provide tools to nurses seeking quality in their nursing care by using indicators that address the NP, it is necessary for nurses to have the standardized and computerized nursing data that are essential to assessing the effectiveness of care and verification of contributions to outcomes achieved by patients. 

Additionally, it is also necessary that studies address the relationship among Nursing Diagnoses (NDx), interventions, and outcomes, showing the contributions made by NP and its importance to the quality of systematizing care^(^ 8 ^)^.

Since SIPETi was developed to generate indicators capable of establishing this relationship, the system's assessment module, from which data can be retrieved, needs to be validated for the system to be used with this purpose. 

This study's general objective was to analyze NP efficacy using indicators generated by software in an adult ICU located in Belo Horizonte, MG, Brazil. The specific objectives included identifying the incidence and prevalence of nursing diagnoses in a group of inpatients in an ICU in Belo Horizonte and identify the rate of risk diagnostic effectiveness and effectiveness rate in the prevention of complications among inpatients included in the study.

## Method

This cross-sectional study was conducted in an adult ICU in Belo Horizonte, MG, Brazil, with patients hospitalized in the first two beds of the ICU during four months of 2013 (from June 28, 2013 to October 26, 2013). This ICU was chosen because its nursing care was already systematized through the NP since 2008. The NP steps are performed daily with all the ICU inpatients. For that, nurses use worksheets they create themselves in a computerized system. The use of software with daily input was initiated for some of the patients during its implementation, as a test. These two beds were chosen because they are separated from the remaining beds within the ICU's physical structure and also due to the need to establish a number of patients that would enable data collection to be performed by the professionals who volunteered for the task together with the researchers, all specifically trained to collect data using the computerized system. 

In the event a patient included in the study was transferred to a bed not selected for this study, his/her monitoring within the ICU would be performed up to discharge, removal to another unit, or death. Patients were excluded if admitted to one of the selected beds but discharged, transferred to another unit, or if they died before the first data collection using the software.

Individual and collective training was implemented to qualify the RNs and nursing students who would feed the SIPETi. Theoretical-practical content was addressed by one of the researchers. Agreement regarding implementation of the NP stages and its documentation in the computerized system was established among the RNs and students through Batista's concordance test. Three RNs and three students were considered able to perform data collection, as they obtained a level of agreement greater than 80%^(^ 9 ^)^.

The RNs and students properly trained took turns daily to register patients and history-taking (at admission), physical assessment, establishment of Nursing Diagnoses (NDx), nursing planning/prescription, and assessment of care provided to the patients admitted to the first two beds in the ICU up to discharge, transfer, or death. After information was included in SIPETi, data necessary for the computation of nursing indicators were retrieved.

NDx concerning Risk for constipation, Risk for acute confusion, Risk for imbalance in body temperature, Risk for impaired skin integrity, and Risk for falls and the respective actions prescribed during the entire hospitalization, within the studied period, were selected for the calculation of indicators. These NDx were chosen because studies show these are among the most frequent NDx presented by critical patients admitted in ICUs^(^ 10 ^-^ 11 ^)^. Additionally, falls and pressure ulcers have been mentioned as the main events to be prevented for the safety of patients^(^ 12 ^)^ used to compose the SIPETi database, as well as to be used as indicators of the quality of nursing care,.

The following indicators, proposed by the Order of Nurses of Portugal^(^ 13 ^)^, which belong to the set of essential nursing data, were selected:

Incidence:



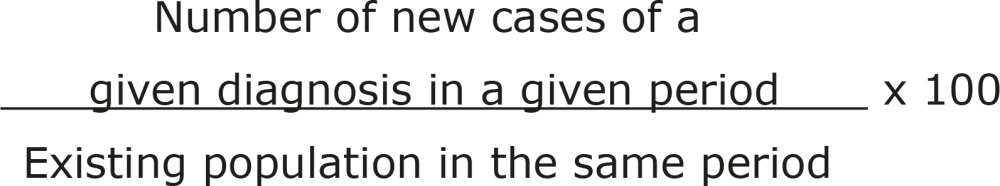



A case concerning a given diagnosis was considered to be new when identified after the patient's first assessment, that is, after admission. Note that a diagnosis under which a patient is admitted is not considered to be a new case, even if the diagnosis is resolved and identified again afterwards. 

Prevalence:



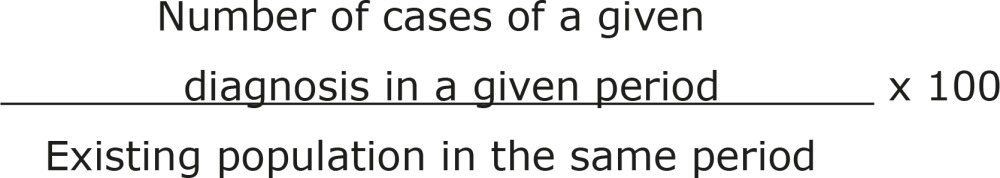



In this study, a case of a given diagnosis was considered when it was identified at some point during the patient's hospitalization.

Rate of risk diagnosis effectiveness:



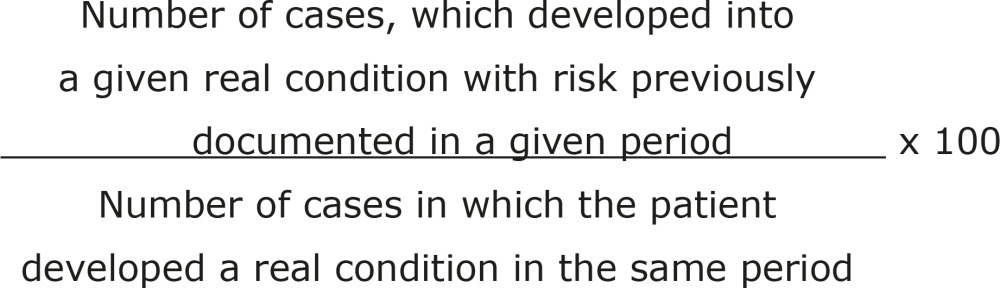



**Figure 1 -:**
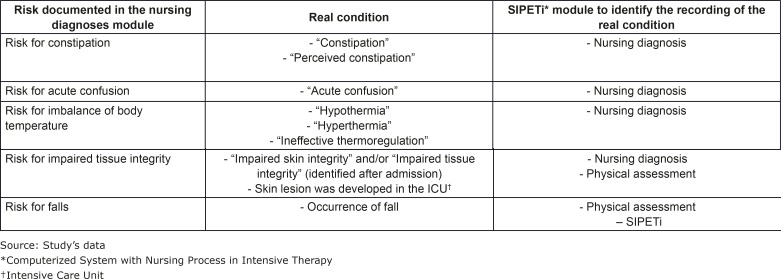
Risk Nursing Diagnoses and respective real conditions identified among the study's patients

The number of cases of patients who developed a real condition was identified based on real nursing diagnoses or adverse events, as described in Figure 1. A case in which risk was previously documented was considered one in which a risk diagnosis was identified on the day before the occurrence of the real condition.

Rate of effective prevention of complications:



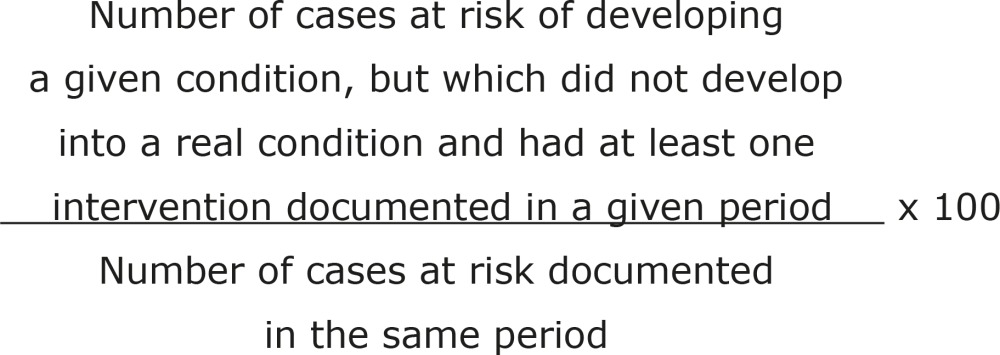



In SIPETi, nursing actions are prescribed for a certain diagnosis, enabling the computation of the rate of effectiveness in the prevention of complications.

Data concerning the calculation of indicators were generated by SIPETi. Percentages were established and the results of these computations are presented in tables. 

## Results

A total of 142 patients were admitted to the ICU during data collection. Seventeen (12%) patients occupied the beds selected for this study and all these composed the study's sample. Ten of these patients were male and represented 59% of the total. Age ranged from 26 to 91 years old, with an average age of 68 years old and a median of 76 years old. All the patients were hospitalized using health insurance plans. Duration of hospitalization ranged from three to 42 days, with 13 days on average and a median of nine days.

The NDx Risk for imbalanced body temperature and Risk for impaired skin integrity were the most incident: 23.53% and 11.76%, respectively. The NDx Risk for acute confusion and Risk for falls had an incidence of 5.88%, while Risk for constipation had an incidence of 0%.

In regard to prevalence, Risk for impaired skin integrity was prevalent in 17 (100%) patients, followed by Risk for imbalanced body temperature and Risk for falls, both prevalent in 15 (88.23%) patients. Risk for constipation was prevalent in 13 (76.47%) patients, while the least prevalent was Risk for acute confusion; only two (11.76%) patients were affected by it.

We verified that Risk for constipation and Risk for impaired skin integrity obtained the highest rate of diagnostic accuracy, that is, all the patients who developed constipation or skin lesions, were previously diagnosed with Risk for constipation and Risk for impaired skin integrity, respectively. 

The rate of diagnostic effectiveness for Risk for imbalanced body temperature was 85.7% and 0% for Risk for acute confusion. The rate of diagnostic effectiveness was not established for Risk for falls because the event did not occur. 

Data concerning the calculation of the rates of risk diagnostic effectiveness are presented in Table 1.

**Table 1 -:** Rate of diagnostic effectiveness concerning the diagnoses addressed in this study. Belo Horizonte, MG, Brazil, 2013

Nursing diagnoses	Number of cases that developed to a real condition	Number of cases that developed real conditions with risk previously documented	Rate of risk diagnostic effectiveness (%)
Risk for confusion	03	0	0
Risk for constipation	03	03	100
Risk for imbalanced body temperature	06	07	85.7
Risk for impaired skin integrity	10	10	100
Risk for falls	0	0	-

The rate of effectiveness in the prevention of complications such as acute confusion and falls was 100%. The same indicator was observed for the NDx Risk for constipation, Risk for imbalanced body temperature, and Risk for impaired skin integrity: 76.9%, 73.4% and 70.6%, respectively. Data used for calculating rate of effectiveness in the prevention of complications for the diagnoses selected for this study are presented in Table 2.

**Table 2 -:** Rate of effectiveness in the prevention of complications concerning the diagnoses addressed in this study. Belo Horizonte, MG, Brazil, 2013

Nursing diagnoses	Number of cases at the risk of complication	Number of cases at the risk of complication but which did not developed and actions prescribed	Rate of effectiveness in the prevention of complications (%)
Risk for acute confusion	02	02	100
Risk for constipation	13	10	76.9
Risk for imbalanced body temperature	15	11	73.4
Risk for impaired skin integrity	17	12	70.6
Risk for falls	15	15	100

## Discussion

The NDx Risk for constipation was not identified during the stay of any of the patients, though it obtained high prevalence. This shows that all the patients had the Risk for constipation identified at the time of admission. This finding is due to the fact that NDx have risk factors commonly found in patients admitted to ICUs, such as insufficient physical activity, the use of sedatives and opioids, and vasopressor drugs, among others^(^ 14 ^)^. Another study also identified this NDx in most patients hospitalized in an adult ICU^(^ 15 ^)^.

The NDx Risk for falls had low incidence because it was identified in only one patient during hospitalization. Its prevalence, however, was high. This means that Risk for falls was identified in most patients at the time of admission. This finding is due to the fact that the risk factors of this specific ND are common among patients admitted in the studied ICU, such as being older than 65 years old, acute disease, and impaired physical mobility.

The NDx Risk for acute confusion had low incidence and prevalence. These indexes can be associated with the fact that some of the factors related to acute confusion include being older than 60 years old and having dementia, while the latter is often confounded with acute confusion itself, leading to errors of omitting the NDx. Since the diagnostic possibilities presented by SIPETi result from mapping evidence included in the physical assessment module, neither advanced age nor dementia are mapped in the NDx module because they are documented in the registration and history-taking modules. The fact that SIPETi still does not allow adding a nursing diagnosis that is not mapped to constant changes found in physical assessments may explain the low incidence and prevalence of this ND.

The NDx Risk for imbalanced body temperature and Risk for impaired skin integrity were identified in four patients after admission, constituting an incidence of 23.53%. Both presented high prevalence because they were predicted for 15 (88.23%) and 17 (100%) patients, respectively. This contrast between low incidence and high prevalence for both NDs may be explained by the fact that patients admitted to an ICU usually present risk factors for these NDx. Risk for imbalanced body temperature presents risk factors such as extreme age, inactivity, sedation, changed metabolic rate, medications that cause vasoconstriction and vasodilatation, among others, which are common in severely ill patients^(^ 14 ^)^. One study shows that risk factors such as immobility and mechanical factors are among the risks most frequently found concerning Risk for impaired skin integrity among patients hospitalized in ICUs^(^ 16 ^)^. The high prevalence of the NDx Risk for impaired skin integrity corroborates data found in another study, which also predicted this ND for 100% of the women hospitalized in an ICU^(^ 17 ^)^.

Three patients were identified with the ND Acute confusion, though none of them presented the Risk documented on the previous day, explaining a rate of diagnostic accuracy of 0%. This finding may also be explained by the same reason that was a possible explanation of the low incidence and prevalence of this ND; that is, the omission of an ND due to a failure in the process of investigation and diagnosis and/or the fact that this ND does not appear to be a diagnostic possibility because some of its risk factors are included in the registration and history-taking modules, neither of which are included in the NDx module.

Three patients were diagnosed with Intestinal constipation (none with perceived constipation), but all had the ND Risk for constipation identified on the day before the identification of the condition, translating into a rate of diagnostic accuracy of 100%. This finding, allied with the high prevalence of this ND in the study, reflects the attention nurses have paid to constipation. The occurrence of many risk factors for this diagnosis such as those of a pharmacological, functional, physiological, mechanical, and psychological nature, allied with a characteristic that is common among patients in ICUs, being bedridden and a consequent reduction in physical activity, may explain why nurses easily identify patients at risk. Another piece of information that contributes to this assertion is the fact that this ND presented an incidence of 0%; that is, it was not identified during the hospitalization but at the time most of the patients were admitted.

Six patients were identified with Hypothermia or Hyperthermia, while one presented both during hospitalization; that is, there were seven cases and none of them was identified with Inefficient thermoregulation. Because only in one case did the patient diagnosed with hyperthermia not present documented Risk for imbalanced body temperature, the rate of diagnostic effectiveness was 85.7%.

Five patients were identified with the ND Impaired skin integrity during hospitalization and none of the patients presented Impaired tissue integrity. Among these, 17 patients developed lesions during hospitalization in the ICU. It was established, for the study's purpose, that the number of lesions developed in the ICU associated with ND Risk for Impaired skin integrity or Impaired tissue integrity would be considered the number of cases that developed a given real condition (skin lesion). This is the case because a patient is identified with both ND Impaired skin (or tissue) integrity and Risk for impaired skin integrity. Hence, if a real condition develops, that is, a skin lesion, it may not be identified by identifying the diagnostic title only. Therefore, there is importance in describing in the physical assessment when a skin lesion develops in the ICU so that it is characterized as an adverse event, that is, a real condition.

Additionally, because more than one lesion was identified per patient in just a few days and the indicator of rate of risk diagnosis effectiveness is intended to verify the effectiveness of diagnosing the risk before the condition develops, when a patient was reported as having more than one lesion developing on the same day was considered a single case. Hence, the number of cases considered for the denominator of this indicator was ten. For all these cases, the ND Risk for impaired skin integrity was identified on the day before the lesion developed, reflecting a rate of diagnostic effectiveness of 100%. It is worth noting that the ND Impaired skin integrity was identified for the patients with skin lesions without considering the rupture of skin secondary to the introduction of invasive devices like venous access for instance.

The rate of diagnostic accuracy regarding Risk for falls could not be established since there was no occurrences of falls among the study's patients. It is worth noting, however, that this ND was one of the most prevalent, with 88.23%. One study also showed low occurrence of falls in the ICU, corresponding to 0.05%^(^ 18 ^)^. In addition to a concern regarding the complications this adverse event may cause, falls are less common in ICUs because patients are usually bedridden due to the need for continuous monitoring and are not always in a condition where walking or transferring themselves to an armchair during any given time is possible. Another potential explanation for a lack of falls is due to the fact that nursing actions "to maintain high bed rails" and "to identify the patient's cognitive or physical deficits (drowsiness, visual deficit, psychomotor agitation, mental confusion, immobility or limited physical mobility or paresthesias) that can increase the risk of falls" are among the most prevalent in this study.

The highest rate of prevention of complications observed was related to Risk for Acute confusion and Risk for falls: all the patients diagnosed with these risks received a documented action and did not develop the real condition, in this case, acute confusion or a fall. This finding confirms one of the advantages of using software in the prescription of specific actions for each nursing diagnosis. When nursing diagnoses and interventions are interconnected, they facilitate individualized nursing care^(^ 19 ^)^.

The rate of effectiveness in the prevention of complications for Risk for constipation was 76.9%. Three of the 13 patients identified with this ND developed constipation and ten did not. Even though all had documented actions to avoid constipation, it is known that constipation has various related risk factors that hinder direct nursing care, such as advanced age, restricted manipulation secondary to hemodynamic instability, suspended diet, and history of chronic constipation, among other factors.

The ND Risk for imbalanced body temperature was predicted for 15 patients but 11 did not develop hyperthermia, hypothermia or inefficient thermoregulation, culminating in a rate of effective prevention of complications of 73.4%. A documented action was prescribed to all of the patients to prevent complications, including the monitoring of body temperature every two hours, which was prescribed to all the study's patients. 

The rate of effective prevention of complications was 70.6% for Risk for Impaired skin integrity. Of the 17 patients (100%) identified with this risk, 12 did not develop Impaired skin integrity or Impaired tissue integrity during hospitalization. A total of 17 skin lesions developed among five patients. Of these, 13 were pressure ulcers, while nine developed in a single patient, whose risk to develop pressure ulcers, according to Braden scale, was moderate on the day in which three ulcers were verified and high on the remaining days. 

## Conclusion

Data collected in this study enabled analyzing indicators generated by software in an ICU because the indicators reveal how nurses have identified patients' risks and conditions and planned care in a systematized manner. 

Note that the indicator of rate of risk diagnoses accuracy enabled detecting how efficiently the risk of patients to develop a given condition was evaluated. This indicator reinforces the importance of a holistic nursing approach to patients, especially history-taking and physical assessment, given the identification of patients' risk factors, and consequently, the establishment of preventive actions.

Through the indicator of rate of effective prevention of complications it was possible to identify the effectiveness of preventive actions prescribed to the patients. It is worth noting that the actions must be based on evidence, with strong recommendations from the literature, strengthening nursing care with a scientific basis. 

It is, however, necessary that further studies addressing larger samples and other NDx be conducted in order to enable a more comprehensive assessment of NP efficacy. 

Given the undeniable need to provide tools to nurses and establish strategies to measure and improve the quality of nursing care, it is important that essential data concerning nursing be standardized and computerized to assess the effectiveness of care and how nursing care contributed to the outcomes achieved by the patients. In this sense, it is essential to establish indicators that address NP.

It is not possible to infer which given nursing intervention was decisive in keeping a condition from developing, though they certainly contributed. Further research focusing on the efficacy of actions is needed to prevent the occurrence of complications among patients hospitalized in ICUs and, for that, it is necessary that nurses establish nursing prescriptions for each diagnosis identified among the patients under their care.
